# Overcoming Challenges in Oligonucleotide Therapeutics Analysis: A Novel Nonion Pair Approach

**DOI:** 10.1021/jasms.4c00270

**Published:** 2024-08-19

**Authors:** Yoshiharu Hayashi, Yuchen Sun

**Affiliations:** † Bioanalysis Research Department, CMIC Pharma Science Co., Ltd., Hyogo 677-0032, Japan; ‡ Division of Medicinal Safety Science, National Institute of Health Sciences, Kanagawa 210-9501, Japan

## Abstract

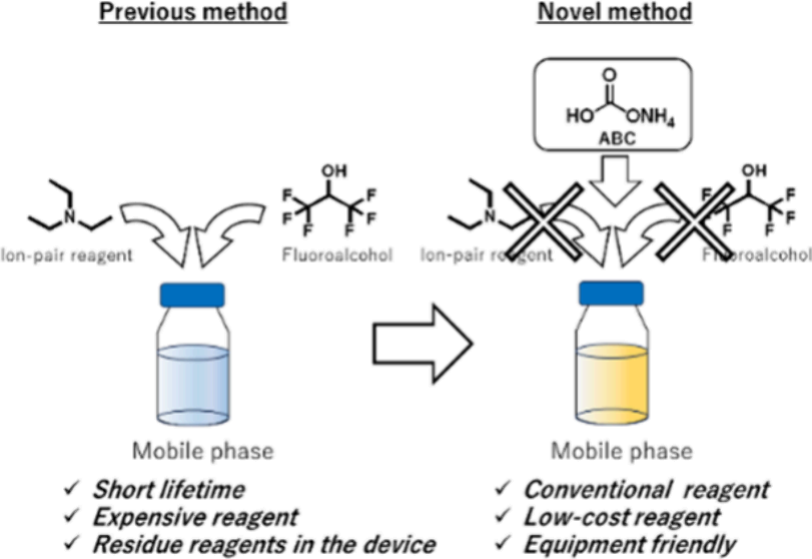

Oligonucleotide therapeutics (OT) have emerged as promising drug modality for various intractable diseases. Recently, liquid chromatography–mass spectrometry (LC-MS) has been commonly employed for characterizing and quantifying OT in biological samples. Traditionally, the ion pairing–reverse phase (IP-RP) LC-MS method has been utilized in OT bioanalyses; however, this approach is associated with several limitations, including the memory effect and ion suppression effect of IP reagents. Therefore, this study aimed to develop a new RP-LC-MS method that eliminates the need for IP reagents. Our investigation revealed that ammonium bicarbonate was essential for the successful implementation of this nonIP-RP-LC-MS-based bioanalysis of OT. Moreover, the developed method demonstrated high versatility, accommodating the analysis of various natural or chemically modified oligonucleotides. The sensitivity of the method was further assessed using reconstituted plasma samples (the lower limit of quantification in this experiment was 0.5–1 ng/mL). In summary, the developed nonIP-RP-LC-MS method offers an easy, reliable, and cost-effective approach to the bioanalysis of OT.

## Introduction

Oligonucleotide therapeutics (OT) have garnered significant attention in recent years.^1−3^ In drug development, the accurate evaluation of the drug concentration in biological samples (bioanalysis) is crucial for assessing both efficacy and toxicity. In this regard, the use of liquid chromatography–mass spectrometry (LC-MS) for the bioanalysis of OT is becoming increasingly common.^2,4,5^

In the analysis of OT using MS, ion pairing (IP)–reverse phase liquid chromatography (RP-LC), which employs alkylamine and fluorinated alcohol, is the mainstream approach.^3^ Nevertheless, this method is fraught with several important concerns. The contamination from IP reagents persists in the LC pump, necessitating a dedicated LC for oligonucleotide analysis using IP reagents and prolonged downtime for cleaning for other analyses using the same instrument. Moreover, the fluorinated alcohol used as an acidic modifier is costly, presenting challenges in terms of both labor and expense.^3,6,7^ To overcome these issues, it is essential to revisit^8^ and refine the nonion pairing RP-LC (nonIP-RP-LC) method for oligonucleotide separation to enhance sensitivity and compatibility with current LC-MS systems.

Therefore, the main purpose of this study is to establish a novel nonIP-RP-LC-MS method that is free from IP reagents for the bioanalysis of OT, with the aim to overcome the analytical limitations posed by conventional approaches that utilize IP reagents.

## Method

The OTs used in this study were chemically synthesized by Ajinomoto Biopharma Services (Osaka, Japan). The structures of each compound are illustrated in Figure S1. The standard samples were prepared by diluting the stock of the OTs (50 or 100 μg/mL) with a mixture of TE buffer and methanol in a 70:30 (v/v) ratio. Biological samples were prepared by diluting the stock solution of OTs with reconstituted plasma, obtained by precipitating proteins from mouse plasma using methanol, drying the supernatant, and redissolving it to its original volume.

The LC-MS system employed combined a Vanquish instrument (Thermo Fisher Scientific, MA, USA) with an Orbitrap Q Exactive Plus instrument (Thermo Fisher Scientific). Samples were separated on a YMC (Kyoto, Japan) 2.1 × 50 mm Accura Triart C18 column (S-1.9 μm, 12 nm) at 85 °C. Mobile phase A consisted of 10 mM ammonium bicarbonate (ABC), and mobile phase B was methanol. ABC (guaranteed reagent grade) was purchased from Kanto Chemical (Tokyo, Japan). The gradient and flow rate conditions are presented in Table S1. MS data acquisition was conducted in positive ion mode after the LC separation of analytes, as it demonstrated superior sensitivity compared to negative ion mode when analyzing dT (Figure S2). The standard samples were measured in Full MS mode, while the biological samples were measured in PRM mode. Details of the MS parameters are listed in Table S1.

The integration of peak areas and construction of calibration curves were performed using Xcalibur and QuanBrowser software (version 4.1; Thermo Fisher Scientific). Peak area was calculated using the most sensitive ion for standard samples (Full MS mode) and three fragment ions (*m*/*z* 111.044, 136.062, 152.057) for biological samples (PRM mode). The mass tolerance in the peak detection process was set at 5 ppm. Lumasiran antisense strand (Lum_AS) with 2′-O-methoxyethyl (MOE) modifications in replacement of 2′-O-methyl (OMe) modifications was used as an internal standard substance (IS). The calibration curves were constructed by plotting the peak area ratio of Lum_AS or lumasiran sense strand (Lum_S) to IS, employing weighted least-squares (1/*x*^2^) linear regression analysis.

## Results and Discussion

### Investigation of NonIP-RP-LC/MS Method with dT

**Figure 1:**
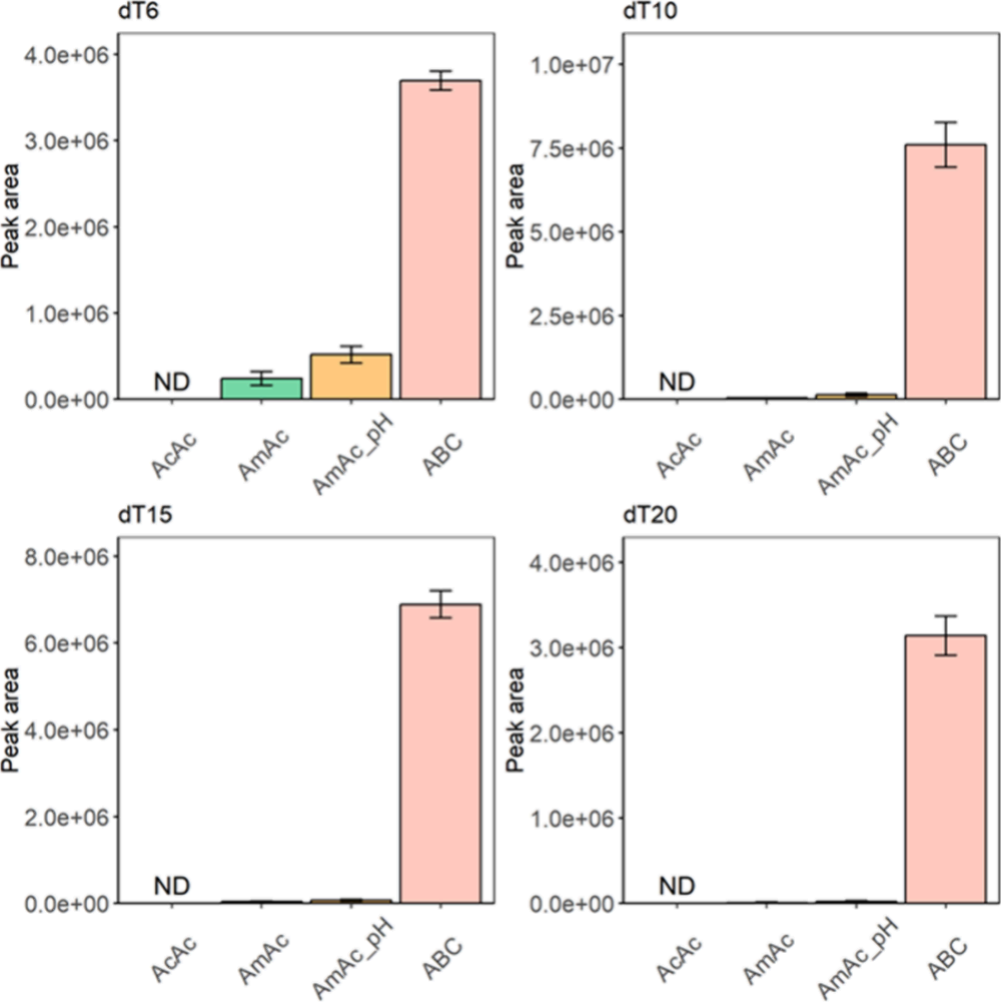
Relationship between the detected peak area of dTs and the mobile phases. Peaks detected in positive ion mode are shown. Data are expressed as mean ± SD (*n* = 3). ND: Not detected, AcAc: 0.1% acetic acid, AmAc: 10 mM ammonium acetate, AmAc_pH: 10 mM ammonium acetate adjusted to pH 8, ABC: 10 mM ammonium bicarbonate.

To develop the nonIP-RP-LC method for OTs, we first optimized the mobile phase conditions using natural polythymidines with different lengths: dT6, dT10, dT15, and dT20 (Figure S1). The retention time (RT) and the detected peak area of each compound in various types of mobile phase A—supplemented with 0.1% (v/v) acetic acid (AcAc), 10 mM ammonium acetate (AmAc) (pH not controlled, pH 6.5), 10 mM AmAc (pH adjusted to 8.0), and 10 mM ABC (pH not controlled, pH 8.0)—were examined. Upon the investigation, methanol was selected as the fixed organic mobile phase. Our data indicated that the mean RT remained nearly constant across AmAc-and ABC-based mobile phases, with dT6 at 3.7 min, dT10 at 4.0 min, dT15 at 4.1 min, and dT20 at 4.2 min (Figure S2), while no detectable peak related to the target dTs was observed for the AcAc-based mobile phase (Figure 1). Contrary to the constant RT, our results demonstrated that the detected peak area of dTs was greatly affected by the type of mobile phase. For all dTs, the mobile phase supplemented with 10 mM ABC exhibited the largest peak area values, ranging from 10 to 300 times higher than those observed using AmAc-based mobile phases (Figure 1).

Traditionally, McFarland et al. showed that nucleic acids can be retained through RP-LC employing AmAc as the mobile phase,^8^ and our current findings replicate their observation. However, insufficient MS sensitivity of AmAc-based mobile phase prompted us to investigate the ABC-based mobile phase which has been used for LC/MS-based small interfering RNA (siRNA) analysis,^9^ resulting in dramatically improved MS sensitivity. It is important to discuss how ABC enables the retention of oligonucleotides in the RP analytical column and why it demonstrates a higher MS sensitivity. Although the detailed mechanism remains unclear, a potential explanation may be attributed to ABC’s thermally degradable properties. It is well-known that ABC can generate highly volatile CO_2_ and ammonia under heated conditions (about 60 °C^10^). Previous papers have demonstrated the association of CO_2_ bubble formation in heated electrospray ionization (ESI) droplets with MS sensitivity using ABC-based mobile phases.^11,12^ The absence of the formation of a CO_2_ bubble in ESI droplets in the AmAc-based mobile phase suggests less progress in droplet miniaturization, potentially explaining the observed MS sensitivity degradation. Additionally, the equilibration of ammonia with ammonium ions in ESI droplets (NH_4_^+^ ⇄ NH_3_ + H^+^) might be another important factor. As the ESI droplet size decreases, the concentration of ammonium ions increases, driving the equilibrium toward a higher ammonia concentration. Since ammonia is a volatile compound, it preferentially vaporizes, leaving protons in the droplet. It is considered that the generated protons can form adducts with oligonucleotides, resulting in the detection of positively charged oligonucleotide ions. For the dTs (dT15 and dT20), this hypothesis is consistent with the finding that scanning in positive ion mode yielded higher MS signals than in negative ion mode (Figures 1 and S3). However, this hypothesis does not apply to short dTs, especially dT6 and dT10. The precise mechanism underlying this phenomenon remains unclear. It is possible that the chemical structure/oligonucleotide length and charge distribution exert a more significant influence than the equilibrium of NH_3_. Overall, our data suggest that ABC may serve as a promising additive for nonIP-RP-LC-MS in OT bioanalysis.

### Application of NonIP-RP-LC/MS Method to Therapeutic Oligonucleotide

**Figure 2:**
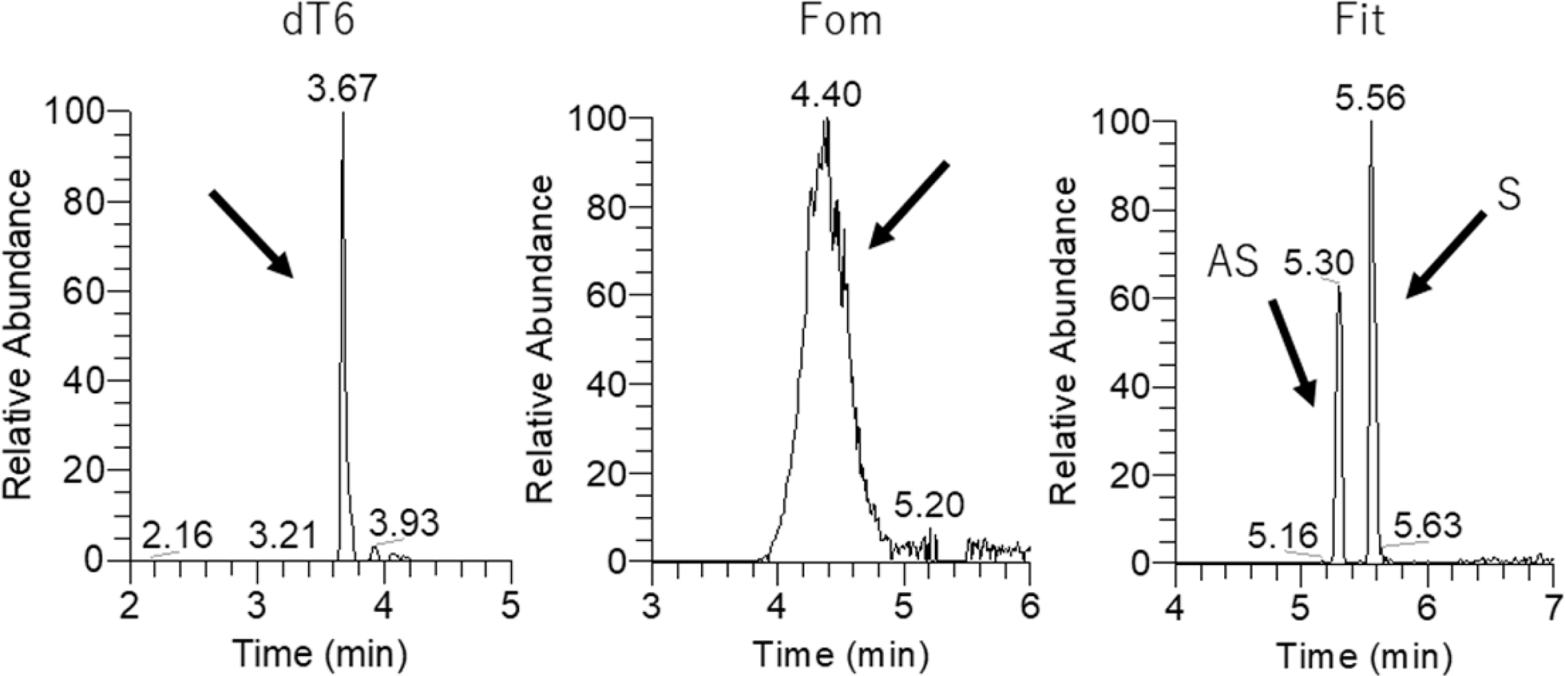
Representative extracted ion chromatograms of various oligonucleotide types in ABC-based RP-LC-MS measurement. AS: antisense strand, S: sense strand. Fomivirsen is a phosphorothioated ASO, and fitusiran is a therapeutic siRNA with partial phosphorothioate modifications. The sample amount of injected oligonucleotides was 2 ng each.

Previous ABC-based LC-MS analyses aimed to measure siRNA in its duplex form,^9^ and the adaptability to other OTs was not clearly evaluated. Therefore, to evaluate the feasibility of the ABC-based approach in OT bioanalysis, we tested the adaptability of this method to various chemically modified OTs, including six antisense oligonucleotides (ASO), three siRNA, and four of their analogs (Figure S1). Representative extracted ion chromatograms are shown in Figures 2 and S4. Importantly, all tested OTs were detected using an ABC-based mobile phase. However, phosphorothioated oligonucleotides exhibited broader peaks compared with those with minimal or no phosphorothioate modifications, likely due to their optical chirality properties, as they form a mixture of many stereoisomers. Therefore, the difference in the peak width was thought to be attributed to the presence of stereoisomers.

Next, the effect of the concentration of ABC ions on RT and peak was examined (Figures S5 and S6). The RT for all compounds increased with the concentration of ABC. Notably, for patisiran antisense strand (Pat_AS), concentrations of 1 and 10 mM ABC were insufficient to retain it on the column, as its RTs (0.17 min at 1 mM and 0.36 min at 10 mM) were close to the theoretical void volume time (0.17 min). At 20 mM ABC, the RT was sufficiently extended, being three times longer than the void volume time (0.87 min). As for the MS intensity, the largest peak area for almost all tested OTs was observed at a concentration of 10 mM ABC (Figure S6). Based on these data, it is recommended that the ABC concentration be optimized between 1 and 20 mM, depending on the retention time and the MS sensitivity of target OTs, when developing the method for OT analysis.

### Evaluation of the Applicability of NonIP-RP-LC/MS Method for Oligonucleotide Bioanalysis

**Figure 3:**
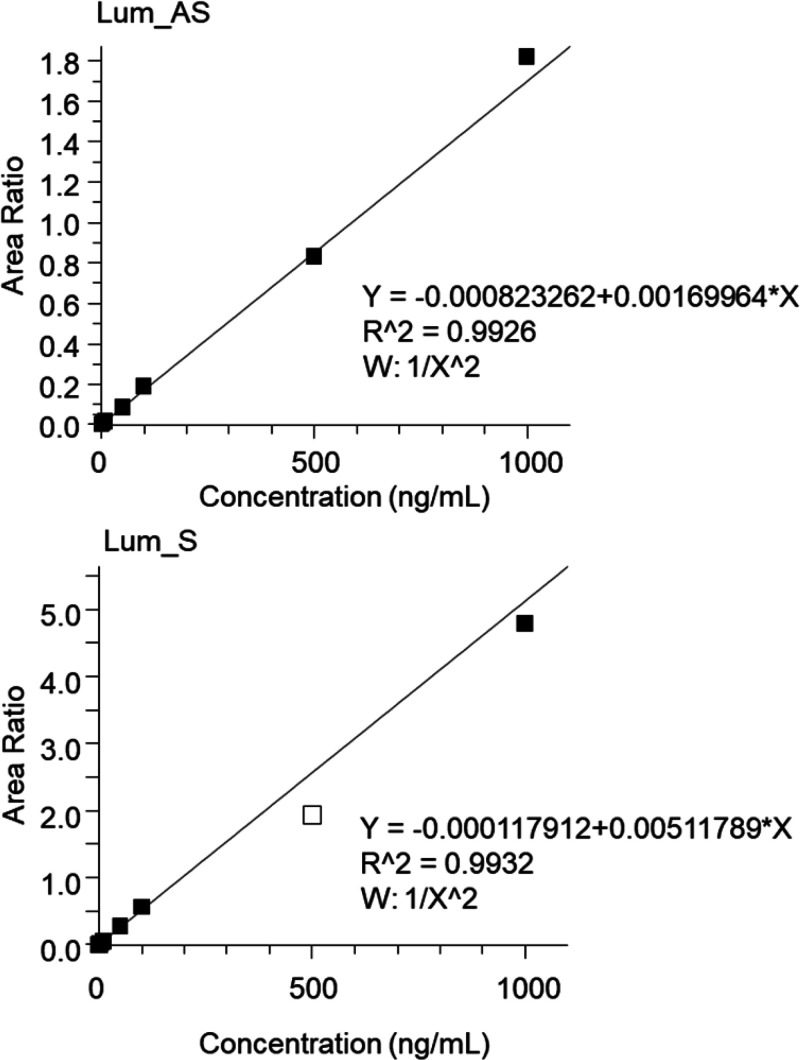
Calibration curve for lumasiran antisense strand and sense strand in reconstituted plasma. Area ratio was calculated as follows: peak area of Lum_AS or Lum_S/peak area of MOE-modified lumasiran. Dynamic ranges were 1–1000 ng/mL (Lum_AS) and 0.5–1000 ng/mL (Lum_S). W: weighting factor. The acceptance criteria for calibration curves were based on ICH M10. The calibration point of 500 ng/mL for Lum_S was excluded from the calculation of the calibration curve because the accuracy of its back-calculation value was below 85%, failing to meet the acceptance criteria.

To assess the method’s adaptability to bioanalysis, we examined the measurement linearity of the current method using reconstituted plasma. In the investigation, Lum was used as a model OT (Figures 3 and S7), and the linearity was examined over a concentration range of 0.1–1000 ng/mL for both AS and S strands using PRM-based MS measurement. As the fragment ions (*m*/*z* 111.044, 136.062, 152.057) produced from the precursor ions (*m*/*z* 1527.04 for Lum_AS, *m*/*z* 1260.59 for Lum_S) displayed relatively high intensity (Figures S8 and S9), these ions were used for calculating the calibration curve. In addition, no interfering peaks were detected at the relevant retention times of Lum_AS and Lum_S in the blank sample (Figure S7). As a result of the measurement of calibration standards, Lum_AS demonstrated good linearity from 1 to 1000 ng/mL (*r*^2^ = 0.9926), and Lum_S from 0.5 to 1000 ng/mL (*r*^2^ = 0.9932) (Figure 3). This broad quantification range and sensitivity are in line with those typically used in OT bioanalysis (Lower limit quantification; approximately 0.2–50 ng/mL).^3^ These findings indicate that ABC-based nonIP-RP-LC/MS is suitable for OT bioanalysis, although further validation based on the guidelines such as ICH M10^13^ is recommended in future studies.

## Conclusion

In this study, we developed a new ammonium bicarbonate (ABC)-based nonIP-RP-LC-MS method devoid of IP reagents for the effective analysis of OTs. The use of ABC as the mobile phase additive in nonIP-RP-LC/MS proved advantageous, with the presence of carbonate and ammonium ions being crucial. Additionally, our data indicate that the ABC-based mobile phase was shown to be applicable to the analyses of various OTs. Using lumasiran as an example, the quantification range and sensitivity achieved with the ABC-based nonIP-RP-LC/MS approach are comparable to those of existing IP-RP-LC/MS, further validating its potential as a reliable bioanalytical method for OTs. Furthermore, the ABC-based nonIP-RP-LC/MS method is expected to offer a more reliable bioanalysis option, potentially overcoming issues related to residual reagents, reagent costs, and instability associated with IP-RP-LC/MS.

## Supporting Information Available

Detailed experimental methods, oligonucleotide sequences and LC-MS data (retention time, peak area, chromatogram, spectra) (PDF)
