# Supplementary material for: Overcoming Challenges in Oligonucleotide Therapeutics Analysis: A Novel Nonion Pair Approach
Source: J Am Soc Mass Spectrom. 2024 Aug 19;35(9):2034–7. doi: 10.1021/jasms.4c00270 (PMC11378278; doi:10.1021/jasms.4c00270)
Supplement: Supplementary file 1 — js4c00270_si_001.pdf [file js4c00270_si_001.pdf]

Supporting Information

Overcoming challenges in oligonucleotide therapeutics analysis: A novel nonion pair approach

Yoshiharu Hayashi[1*]
Yuchen Sun[2]

1 Bioanalysis Research Department, CMIC Pharma Science, Hyogo, 677-0032, Japan
2 Division of Medicinal Safety Science, National Institute of Health Sciences, Kanagawa, 210-9501, Japan

CONTENTS

Table S1. LC gradient conditions........ S2
Table S2. The MS parameters used in this study. S3
Figure S1. Structure of the oligonucleotide therapeutics used in this study. S4
Figure S2. Relationship between the detected retention time of dT and the mobile phases. S5
Figure S3. Relationship between the detected peak area of dT and the mobile phases. S6
Figure S4. NonIP-RP-LC/MS extracted ion chromatograms for various oligonucleotides....... S7
Figure S5. Relationship between the retention time and the concentration of ammonium bicarbonate. S9
Figure S6. Relationship between the peak area and the concentration of ammonium bicarbonate. S10
Figure S7. NonIP-RP-LC/MS extracted ion chromatograms of Lum_AS and Lum_S in the reconstituted plasma. S11
Figure S8. MS spectra for Lum_AS and Lum_S S12
Figure S9. Fragment ions of Lum_AS (z = 5) and Lum_S (z = 7). S13

Table S1. LC gradient conditions.

Gradient 1
(Standard sample) :

Time (min)	Flow rate (mL/min)	A (%)	B (%)
Initial	0.35	98.0	2.0
1.5	0.35	98.0	2.0
10.0	0.35	70.0	30.0
10.1	0.35	5.0	95.0
11.0	0.35	5.0	95.0
11.1	0.35	98.0	2.0
15.0	0.35	98.0	2.0

Gradient 2
(Biological sample) :

Time (min)	Flow rate (mL/min)	A (%)	B (%)
Initial	0.35	90.0	10.0
0.5	0.35	90.0	10.0
5.0	0.35	75.0	25.0
5.1	0.35	5.0	95.0
6.0	0.35	5.0	95.0
6.1	0.35	90.0	10.0
7.0	0.35	90.0	10.0

Table S2. The MS parameters used in this study.

Ionization mode	:	Heated ESI
Sheath gas (unit)	:	50
Aux gas (unit)	:	13
Sweep gas (unit)	:	1
Spray voltage (kV)	:	3.0
Capitally temperature (ºC)	:	269
S-lens RF level	:	80.0
Aux gas heater temperature (ºC)	:	438
In-source CID	:	0.0 eV
Polarity	:	Positive
Full MS		
Scan range	:	*m/z* 800 to 2000
Microscans	:	1
AGC target	:	1e6
Maximum IT	:	500 ms
Resolution	:	140000
PRM		
Microscans	:	1
AGC target	:	2e5
Maximum IT	:	100 ms
Isolation window	:	10 Da
(N)CE / stepped (N)CE	:	NCE 35
Resolution	:	35000
Precursor ions (*m/z*)		
Lum_AS	:	1527.03769 (z=5)
Lum_S	:	1260.58895 (z=7)
Lum_MOE	:	1413.95891 (z=6)

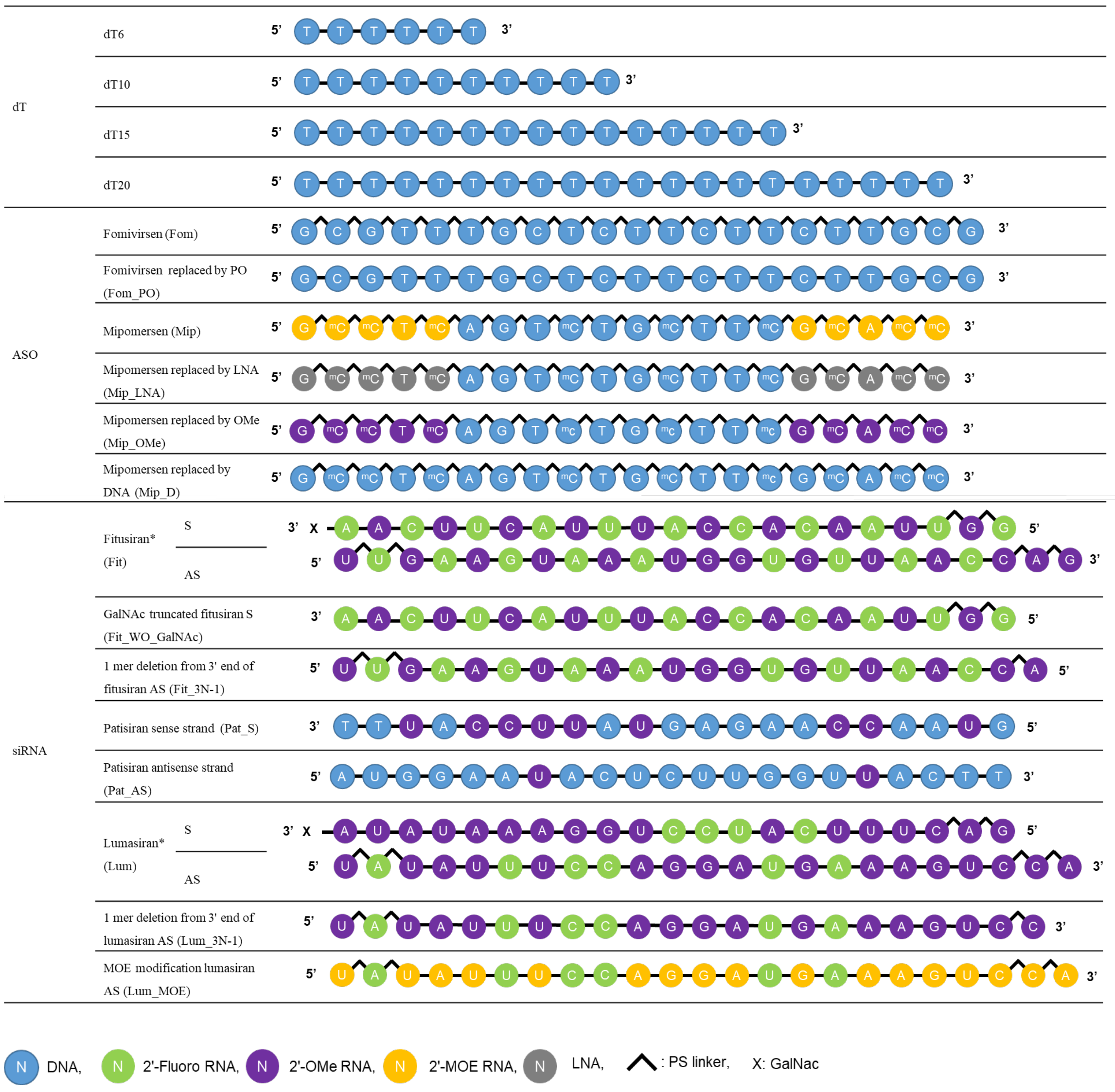

Figure S1. Structure of the oligonucleotide therapeutics used in this study.

*: Indicates a double strand (single strand is assumed unless specified otherwise), S: sense strand, AS: antisense strand.

In this experiment, the fitusiran and lumasiran used different N-acetylgalactosamine (GalNAc) linker structures from the original versions. However, the oligonucleotide sequence and modifications remain identical to those of the original fitusiran and lumasiran.

dT: deoxythymidine, ASO: antisense oligonucleotide, siRNA: small interfering RNA

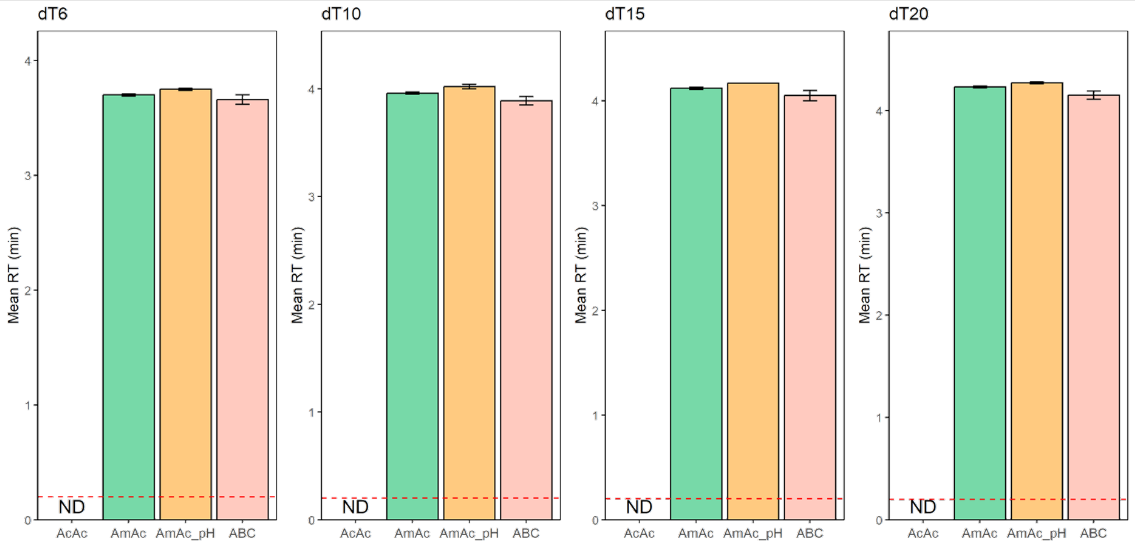

Figure S2. Relationship between the detected retention time of dT and the mobile phases. Data are expressed as mean ± SD (n = 3). ND: Not detected, AcAc: 0.1% acetic acid, AmAc: 10 mM ammonium acetate, AmAc_pH: 10 mM ammonium acetate adjusted to pH 8, ABC: 10 mM ammonium bicarbonate. Red dashed lines denote the void time (approximately 0.17 min).

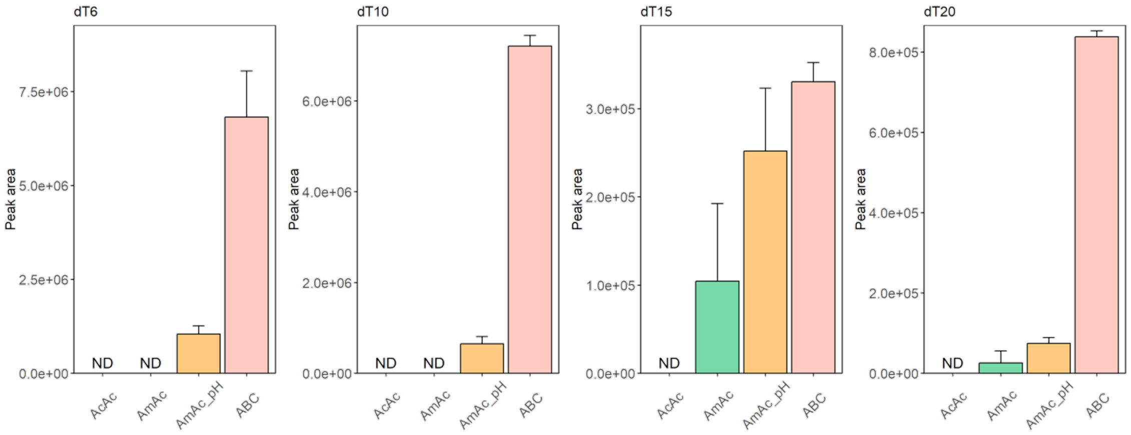

Figure S3. Relationship between the detected peak area of dT and the mobile phases. Peaks detected in negative mode are shown. Data are expressed as mean ± SD (n = 3). ND: Not detected, AcAc: 0.1% acetic acid, AmAc: 10 mM ammonium acetate, AmAc_pH: 10 mM ammonium acetate adjusted to pH 8, ABC: 10 mM ammonium bicarbonate.

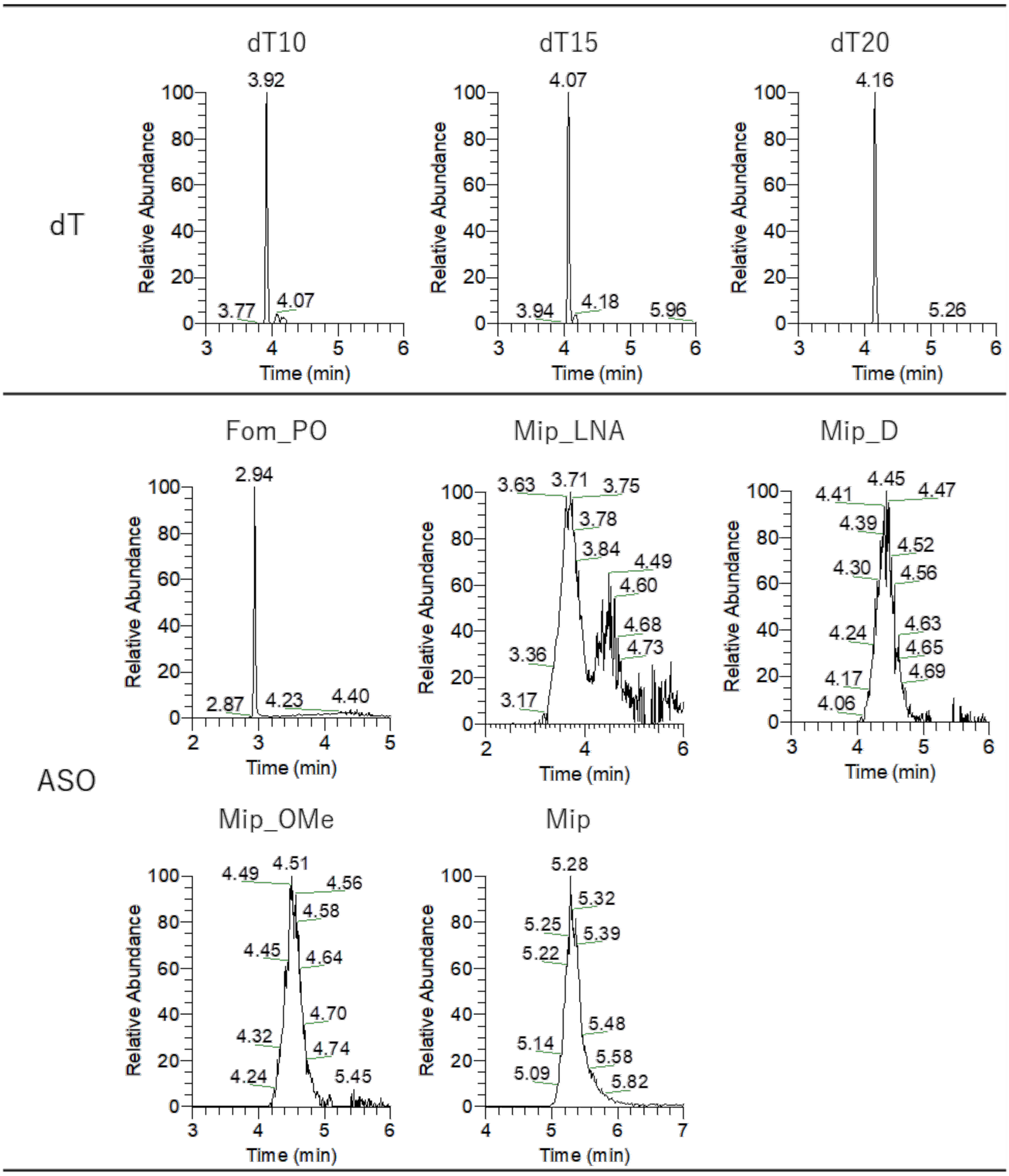

Figure S4. **NonIP-RP-LC/MS extracted ion chromatograms for various oligonucleotides.**

AS: antisense strand, S: sense strand.

On-column amounts were 2 ng for all compounds, except for Pat_AS at 20 ng, and Lum, Lum_3N-1, and Lum_MOE at 4 ng each.

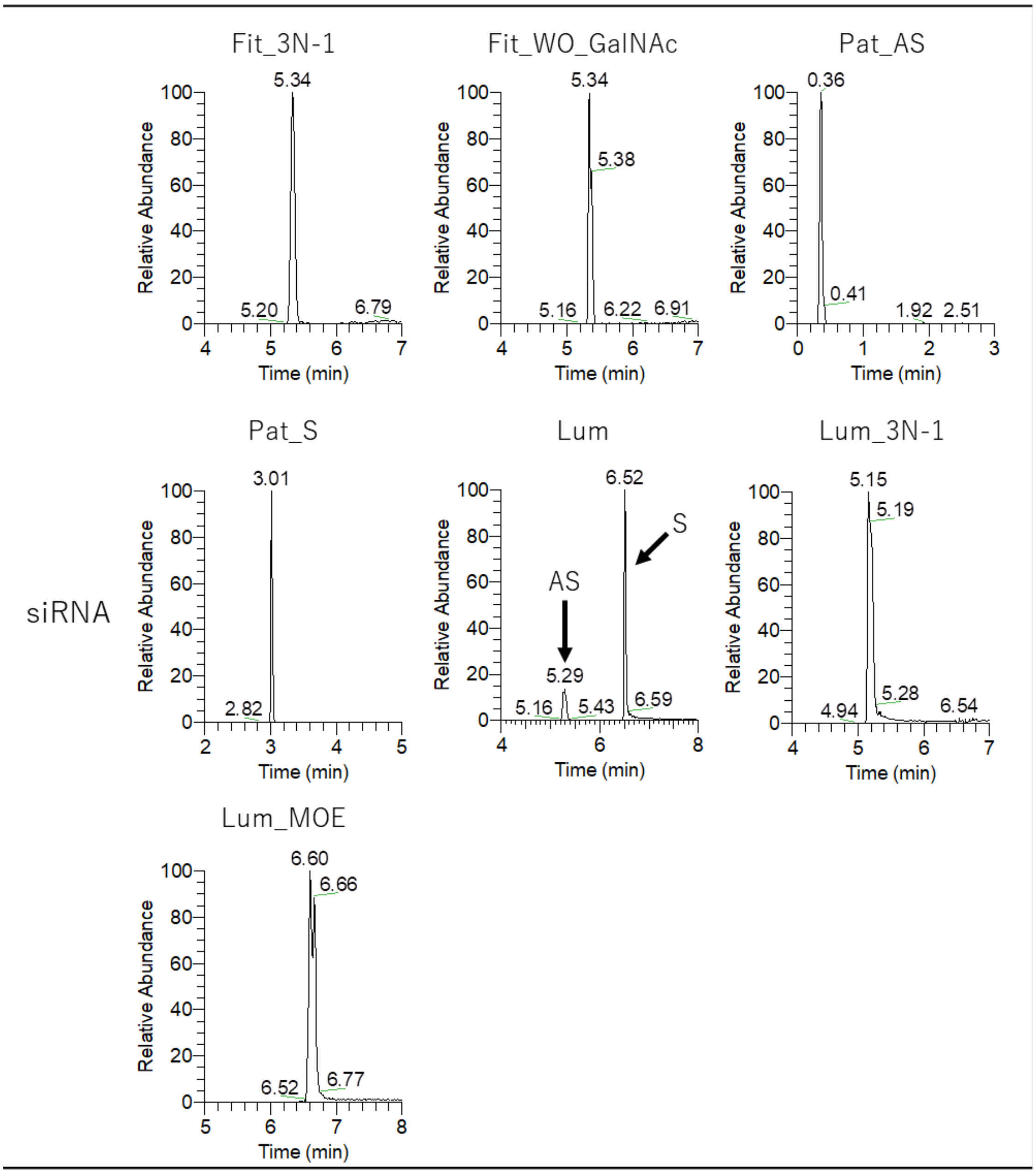

Figure S4. **Continued**

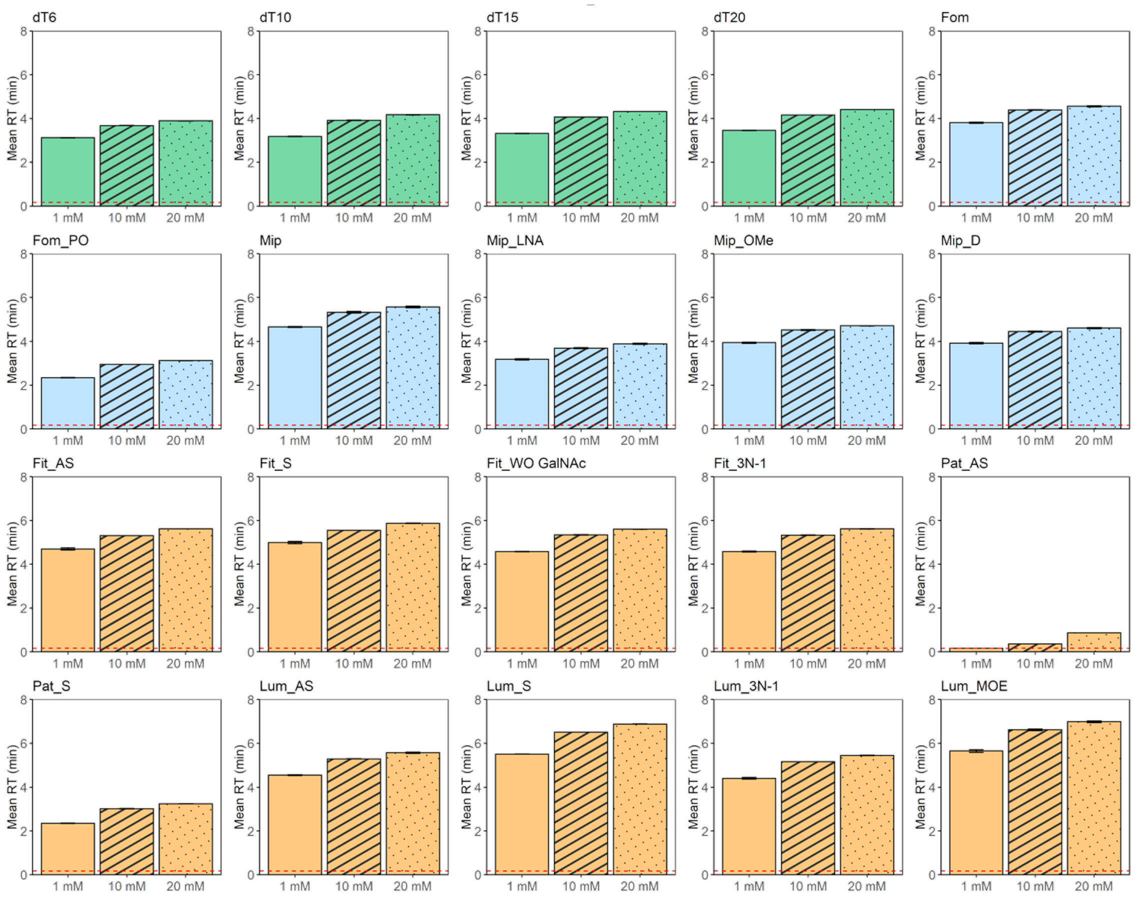

Figure S5. Relationship between the retention time and the concentration of ammonium bicarbonate.

Blue: dT, Green: ASO, Orange: siRNA.

Red dashed lines denote the void time (approximately 0.17 min).

Data are expressed as mean ± SD (n = 3).

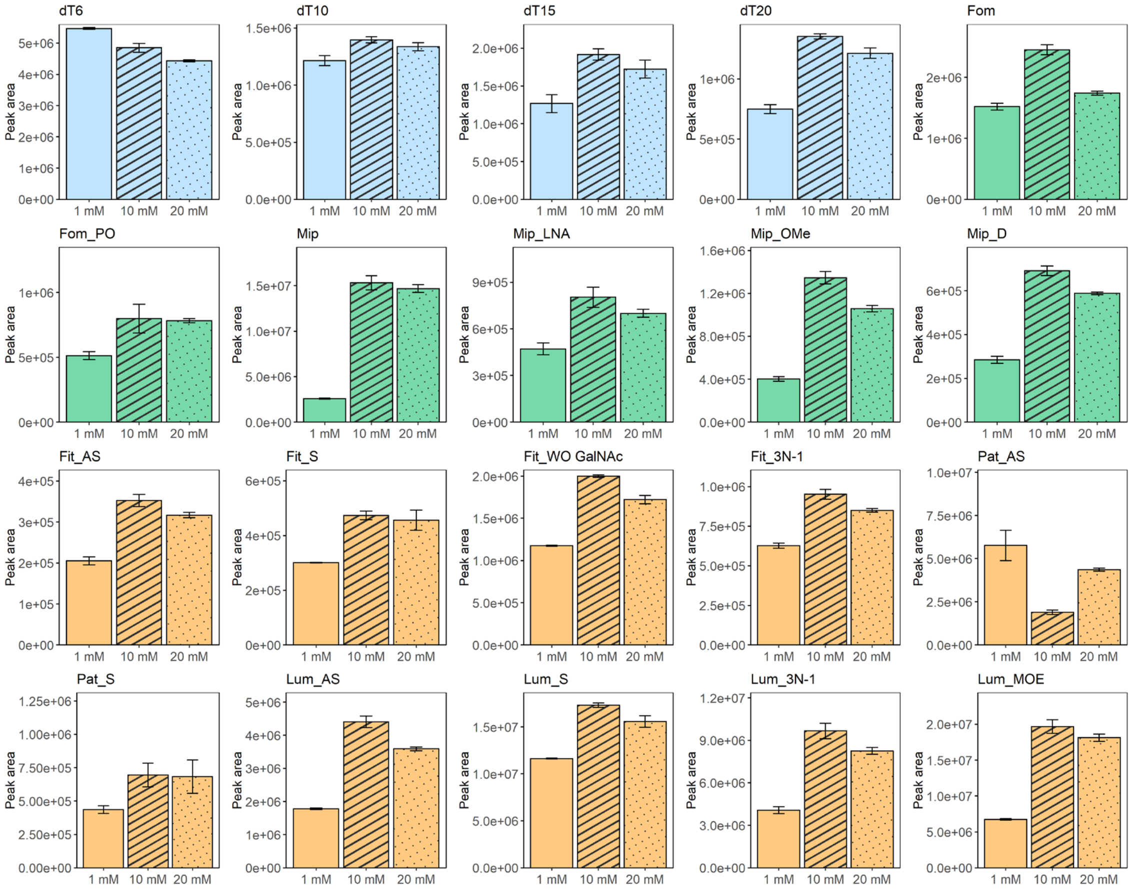

Figure S6. Relationship between the peak area and the concentration of ammonium bicarbonate.

Blue: dT, Green: ASO, Orange: siRNA.

Data are expressed as mean ± SD (n=3).

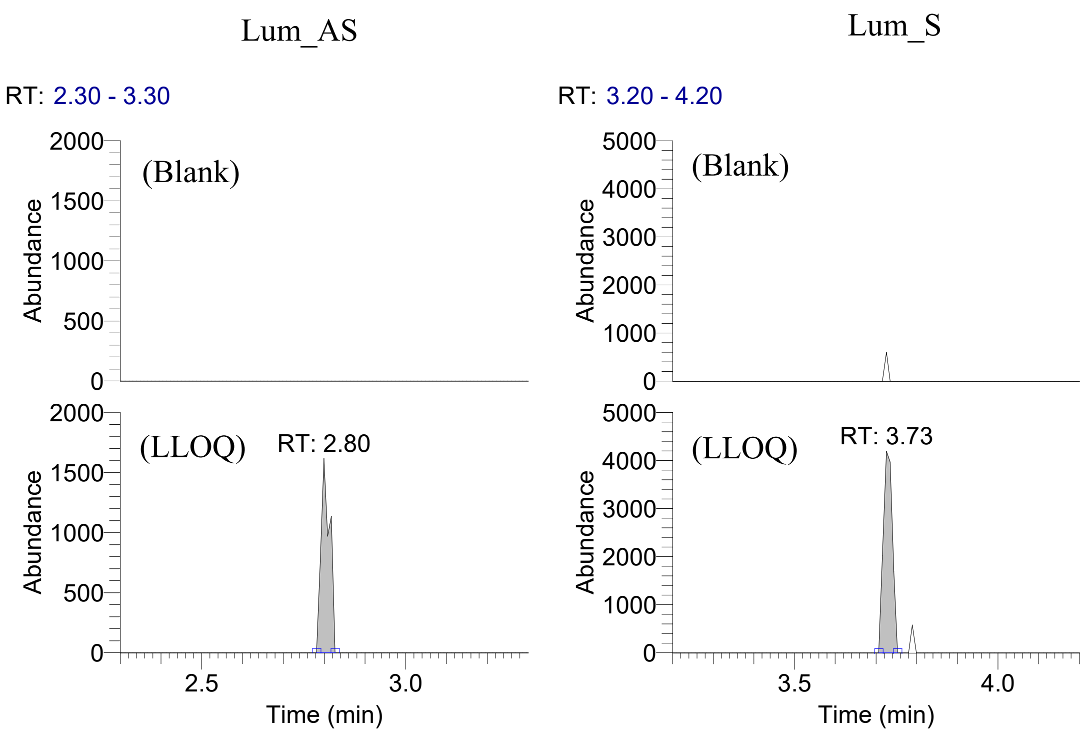

Figure S7. NonIP-RP-LC/MS extracted ion chromatograms of Lum_AS and Lum_S in the reconstituted plasma.

LLOQ: lower limit of quantification

LLOQ was 1 ng/mL for Lum_AS and 0.5 ng/mL for Lum_S, respectively.

Lum_AS

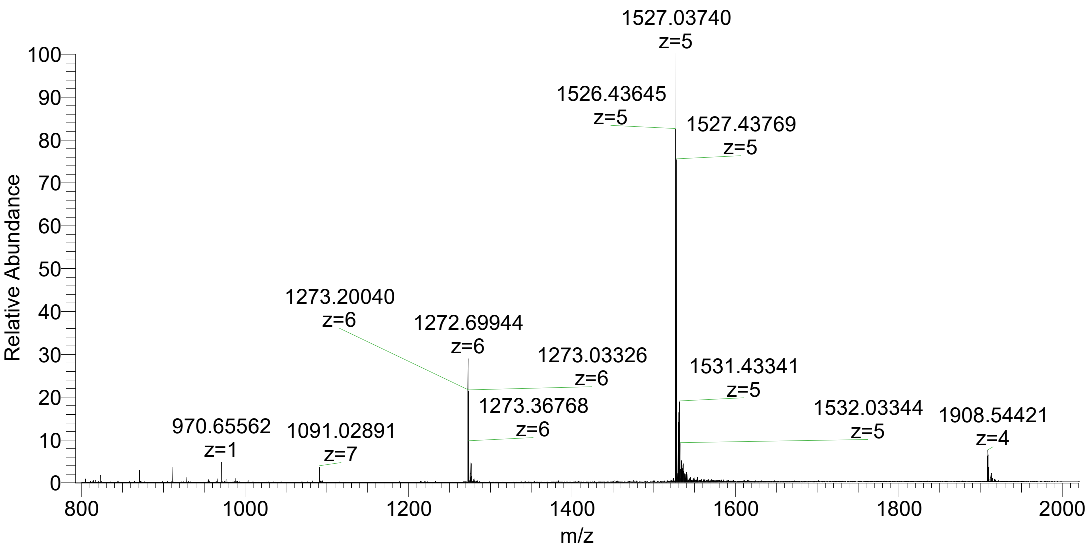

Lum_S

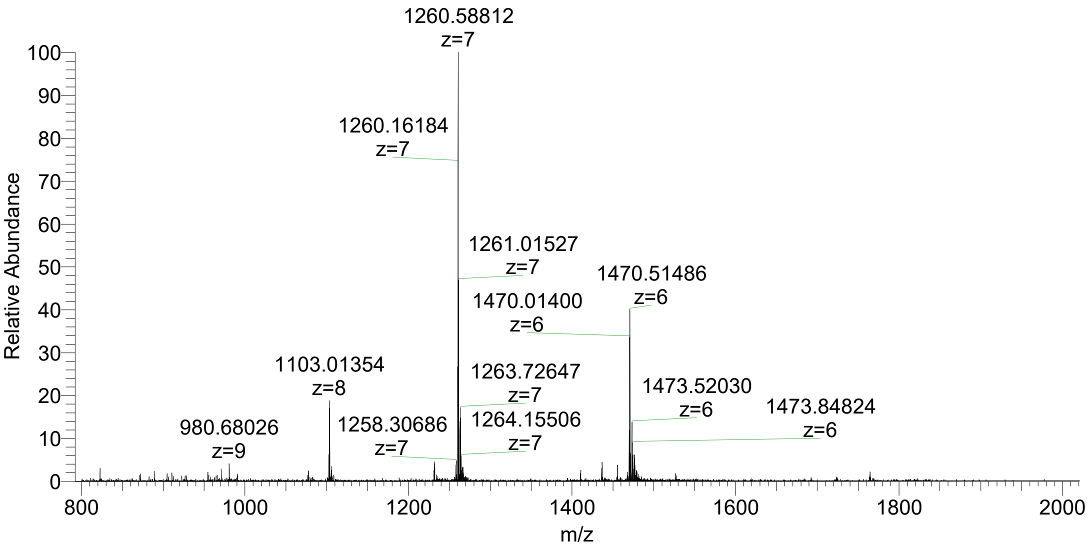

Figure S8. **MS spectra for Lum_AS and Lum_S.**

Lum_AS

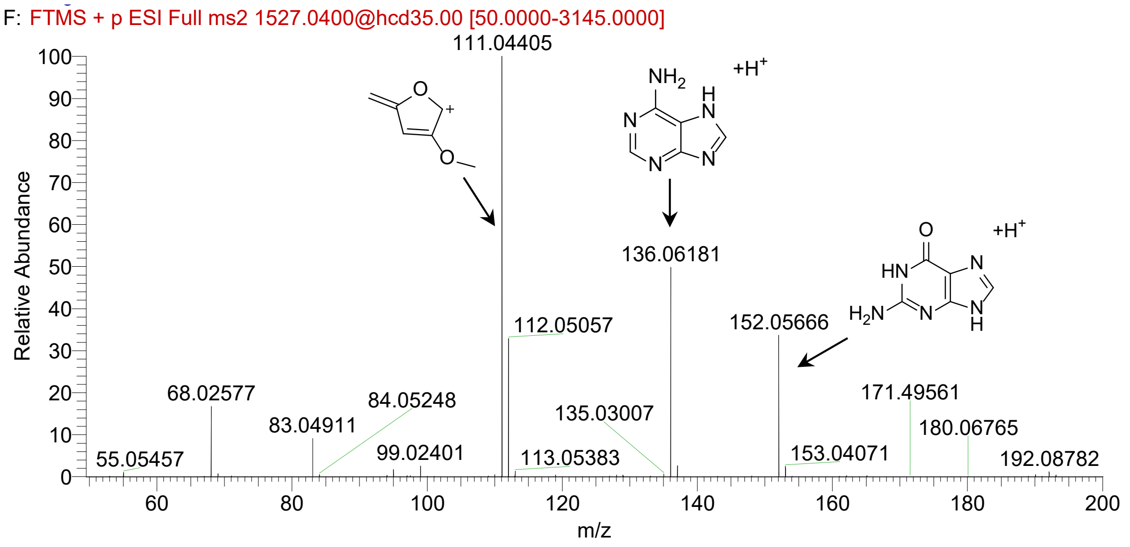

Lum_S

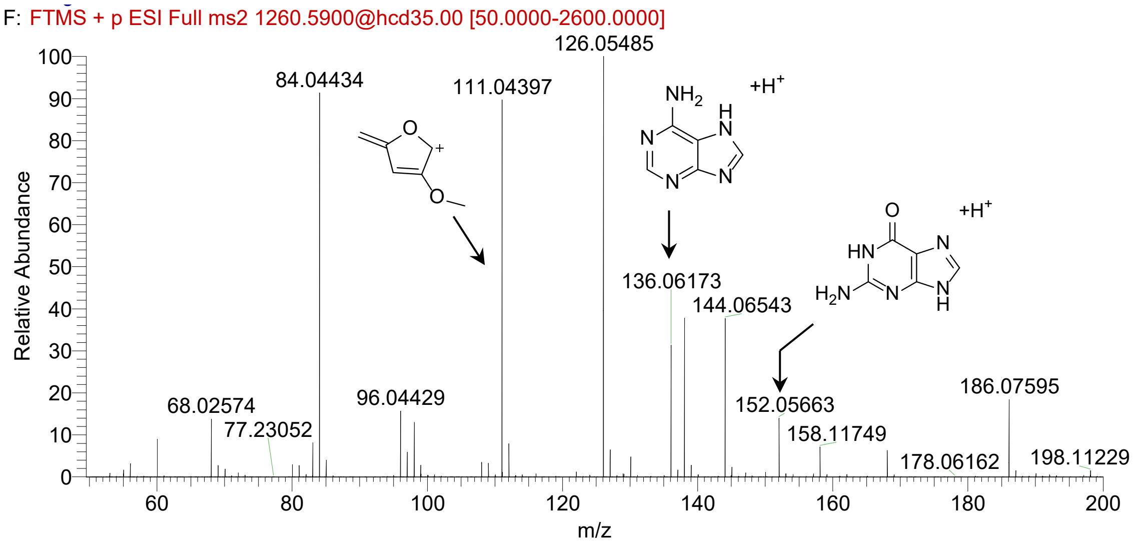

Figure S9. Fragment ions of Lum_AS (z = 5) and Lum_S (z = 7).
